# Improved methods for the detection of histone interactions with peptide microarrays

**DOI:** 10.1038/s41598-019-42711-y

**Published:** 2019-04-18

**Authors:** Christopher J. Petell, Andrea T. Pham, Jessica Skela, Brian D. Strahl

**Affiliations:** 1 Department of Biochemistry and Biophysics, 120 Mason Farm Rd, University of North Carolina at Chapel Hill, NC Chapel Hill, 27599 USA; 2 UNC Lineberger Comprehensive Cancer Center, 450 West Drive, University of North Carolina at Chapel Hill, NC Chapel Hill, 27599 USA

**Keywords:** High-throughput screening, Analytical biochemistry

## Abstract

Histone post-translational modifications contribute to chromatin function largely through the recruitment of effector proteins that contain specialized “reader” domains. While a significant number of reader domains have been characterized for their histone binding specificities, many of these domains remain poorly characterized. Peptide microarrays have been widely employed for the characterization of histone readers, as well as modifying enzymes and histone antibodies. While powerful, this platform has limitations in terms of its sensitivity and they frequently miss low affinity reader domain interactions. Here, we provide several technical changes that improve reader domain detection of low-affinity interactions. We show that 1% non-fat milk in 1X PBST as the blocking reagent during incubation improved reader-domain interaction results. Further, coupling this with post-binding high-salt washes and a brief, low-percentage formaldehyde cross-linking step prior to the high-salt washes provided the optimal balance between resolving specific low-affinity interactions and minimizing background or spurious signals. We expect this improved methodology will lead to the elucidation of previously unreported reader-histone interactions that will be important for chromatin function.

## Introduction

Histone post-translational modifications (PTMs) are integral to the regulation of all DNA-templated functions, most notably gene expression^1,2^. A major mechanism by which histone PTMs contribute to chromatin-mediated regulation is through the interaction of effector proteins (either alone or in the context of a protein complex) with their cognate histone PTMs^3,4^. The interaction these effectors have with their histone PTMs are thought to either confer the specificity of chromatin-associated proteins or complexes to specific regions of the genome or can cause allosteric regulation of the associated protein or complex^5,6^. The importance of reader domain interactions have in chromatin function is underscored by evidence that these domains are frequently mutated in a wide range of human diseases, including cancer^7,8^.

A crucial tool in the evaluation of histone PTM interactions has been the use of histone peptide microarrays that contain synthetic histone peptides that mimic various combinations of histone tail regions and modifications^9,10^. Histone microarrays have been widely used given that they are readily available, can accommodate a large number of differentially modified peptides, can be read by a variety of imaging programs, and are a robust platform where buffer conditions and wash steps can be easily added or modified^11–13^.

While peptide microarrays are a prominent tool in the dissection of reader domain-histone interactions, they also have specific limitations. For example, and in the case of peptide microarrays generated on solid surfaces (SPOT arrays), this platform creates high density peptide libraries through synthesis of the peptides themselves on nitrocellulose membranes^14^. While it is relatively easy to quickly generate a high-density combinatorial PTM library, this platform makes determining the purity and sequence accuracy of the immobilized peptides extremely challenging^14,15^. Additionally, SPOT arrays are limited in the length of peptides that can be accurately synthesized and the peptides on these membranes may have limited rotational freedom^16^. On the other hand, glass slides utilizing streptavidin coating to immobilize biotinylated peptides overcomes many of these limitations, including the ability to immobilize long peptides that have been carefully analyzed by mass spectrometry for accuracy, in addition to the ability to detect peptide interactions with highly sensitive fluoresce detection methods^12^. While glass slide immobilization has many advantages, this platform also has its unique limitations through the finite binding capacity of the streptavidin coating on these slides (for PolyAn slides, ~ 50 fmol/mm^2^ can be immobilized on a typical high capacity streptavidin-coated slide). In our experience, peptide interactions greater that 30 µM are typically missed. Note that SPOT arrays are capable of printing more peptide at a given location, which may give this platform some advantage in low affinity reader domain interactions; albeit with the difficulties mentioned above that make detection, signal variation and background a significant issue^14,15^.

Due to the advantages of glass slide immobilization, we sought to determine if we could further improve the range of detection of this platform, but still maintain all of the advantages the glass slide platform has to offer. Here, we show using a combination of different blocking buffers, salt concentrations, and formaldehyde fixing techniques that the range and signal quality of the peptide microarray platform can be further improved. We show that incubation steps using 1 X PBST with 1% non-fat milk, along with post-binding washing using 1 X PBS with 500 mM NaCl substantially reduced background. Importantly, we found that including a short, low-percentage formaldehyde cross-linking step was able to secure weak affinity interactions while preserving the background reduction from the high-salt wash steps. Together, these steps were able to be combined into a protocol that was able to resolve the interactions of multiple reader domains where past peptide arrays methods missed weaker interactions observed via peptide pulldowns. These improvements improve the robustness of the glass slide platform and should serve to help further elucidate the binding events of poorly characterized reader domains.

## Results

### Histone peptide microarrays do not capture all possible reader domain interactions

**Figure 1:**
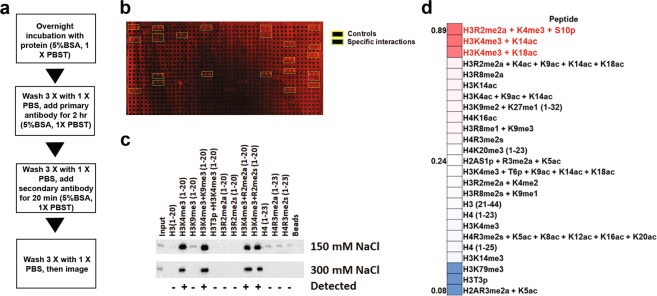
Peptide microarrays do not fully capture interactions detected by peptide pulldowns. (**a**) Flow chart for peptide microarray method. (**b**) Peptide microarray results with the DIDO1 PHD finger. Interactions observed for positive controls (IgG) and peptides are boxed in as indicated. (**c**) Heatmap diagram of top, middle, and bottom hits as sorted by values from quantification by the array for the DIDO1 PHD and with visible hits in red text. (**d**) Examination of the DIDO1 PHD finger in solution peptide pulldowns, using the indicated peptides as selected from array results and previous literature. Images are representative of array results for greater or equal to four experiments (i.e., n ≥ 4 subarrays). Heatmap represents the averages for indicated peptides as derived from the replicates of the array results. The average standard deviation for any given peptide was less than 10% and additional representative array images are also shown in Sup. Fig. S2c. Image of peptide pulldowns is representative of three pulldown experiments.

Histone peptide microarrays have played an essential role in the elucidation of the histone interacting preferences of chromatin reader domains, but are limited in their ability to detect low affinity protein interactions^17,18^. This is especially true for microarrays fabricated on streptavidin-coated slides that have a finite biotin binding capacity. To determine the potential to improve the range of detections for these microarrays, we explored the impact of changing hybridization, cross-linking and washing conditions on a canonical reader domain from DIDO1 (Death-Induced Obliterator). DIDO1 has a critical function in gene regulation and contains a single PHD finger that interacts with H3K4me3 (~1 µM)^19^. With the previous histone peptide microarray protocol, the DIDO1 PHD finger detected a wide range of visible histone interactions, which, in agreement with previous reports, encompassed predominantly peptides carrying H3K4me3 (Fig. 1a,b, green boxes)^19–21^. In addition, and consistent with previous findings, these analyses confirmed the known cross-talk of DIDO1 interactions, which include the ability of H3T3 phosphorylation and H3T6 phosphorylation to abrogate H3K4me3 binding; further, we were also able to recapitulate data showing that H3R2 methylation does not negatively affect H3K4me3 recognition (Fig. 1c and Sup. Fig. S1)^21^. Intriguingly, computational analysis of the peptide arrays using ImageQuant TL or Genepix 5.0 software permitted further detection of several H3 and H4 N-terminal arginine methylation-specific peptides that were close to background levels (Fig. 1b,d, Sup. Fig. S2c).

Given several novel histone interactions were detected for the PHD finger of DIDO1 (e.g., H3R2 and H4R3 methylated peptides), we next performed solution-based peptide pulldown assays to determine if these interactions could be validated. As expected, and at two different salt concentrations, these pulldown assays showed that H3K4me3 is the primary PTM on H3 that is bound by the PHD finger of DIDO1 (Fig. 1c). Intriguingly, they also confirmed the interaction of DIDO1 with several of the N-terminal H4 peptides that were observed, albeit weakly, on the arrays, including interactions with the unmodified H4 N-terminus, H4R3me2a and H4R3me2s (Fig. 1d). Significantly, and at the lower salt concentration (150 mM NaCl), several histone interactions with DIDO1 were detected by peptide pulldowns that were not observed on the arrays (e.g., interactions with several methylated arginine species for H3 and H4) (Fig. 1b,d). These data reinforce the observation that peptide pulldowns are generally more sensitive in the detection of weak histone interactions that are below the threshold for detection on the peptide microarrays.

In order to explore the potential to improve the detection limits of the peptide microarray platform, we devised several parallel and overlapping strategies to tackle three distinct problems that additively contribute to reduced reader domain detection: 1) high background, 2) detection of non-specific binding events, and 3) the inability to detect weak and/or more transient interactions. We reasoned that slight improvements in each of the aforementioned problems would greatly increase the signal-to-noise ratio and enhance the resolution of low-affinity interactions on the histone peptide microarray platform.

### Optimization of the initial blocking conditions

**Figure 2:**
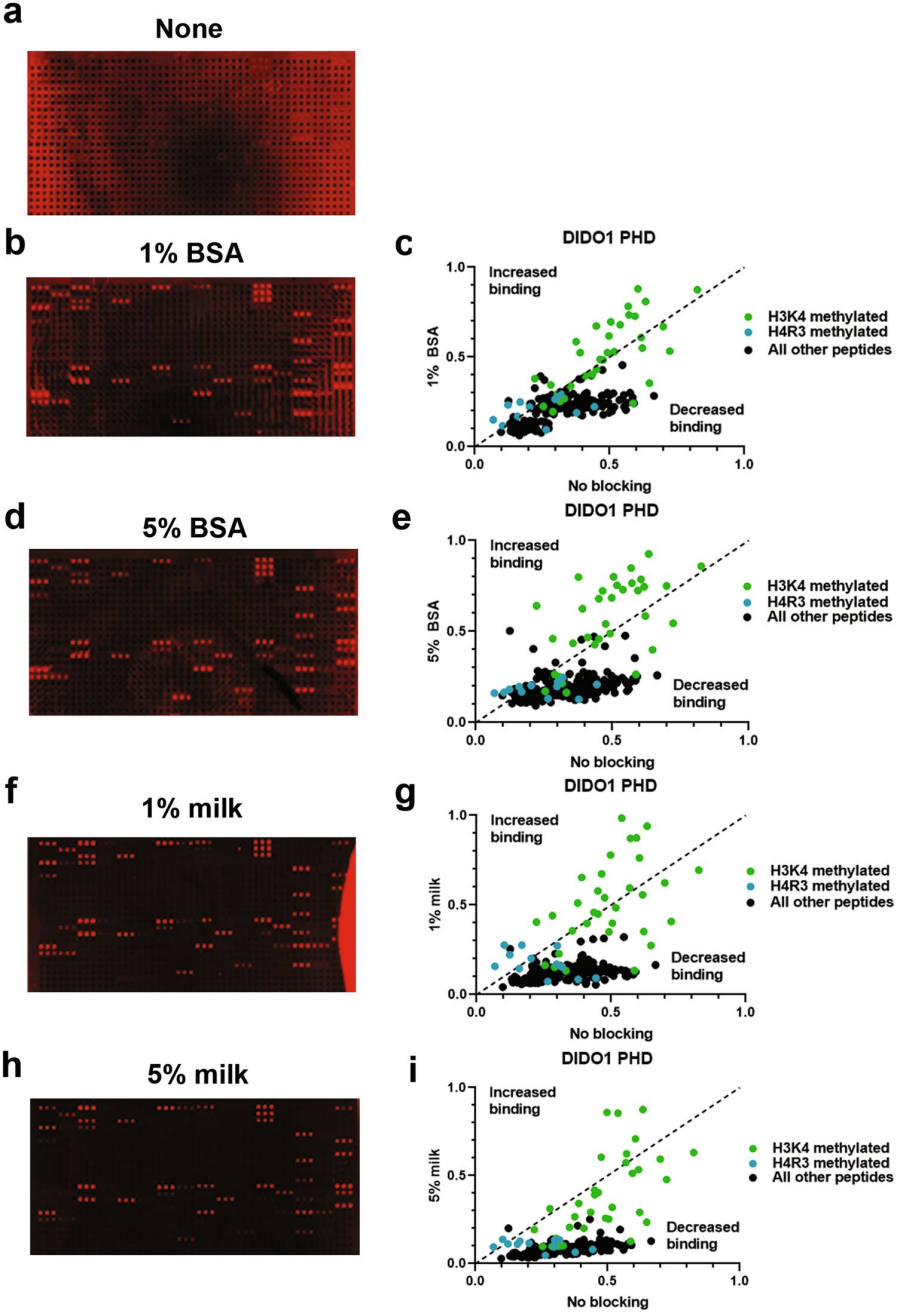
Peptide microarray background is reduced when incubation steps are in 1% milk 1 X PBST. (**a**) DIDO1 PHD array without blocking during incubation steps. (**b–e**) A DIDO1 PHD array carried out in the presence of 1% (**b**) or 5% (**d**) BSA in 1 X PBST during incubation steps that were then quantified by normalizing to a 0–1 scale as described in Methods and compared to no blocking (**c** and **e**, respectively). (**f–i**) DIDO1 PHD array carried out in the presence of 1% (**f**) or 5% milk (**h**) in 1 X PBST during incubation steps that were then quantified and compared to no blocking (**g** and **i**, respectively). Array intensities were linearly adjusted to equivalent levels and are comparable to each other in the figure. Images are representative of array results for greater or equal to four experiments (n ≥ 4 subarrays); for additional representative results see Sup. Fig. S2a–e S2. For quantification, averages are shown for each peptide as derived from the replicates of the array results. The average standard deviation for any given peptide was less than 10%.

We first sought to reduce the visible background typically associated with reader domain hybridization on microarrays by altering the blocking conditions. The blocking conditions previously employed for reader domains and antibodies include 5% BSA in 1 X PBST. To determine the impact of different blocking conditions, we used different amounts of BSA or non-fat dry milk (1% or 5% of either) during the initial incubation steps of the array protocol (see Methods and Fig. 1a). Our experiments found that, in comparison to arrays without a blocking agent, non-fat dry milk served to reduce background better than BSA (Fig. 2 and Sup. Fig. S2a–e). Moreover, 1% non-fat milk produced slightly better images compared to 5% milk. This is supported by quantitative analysis that shows that, while all blocking methods reduced background binding compared to no blocking agent (Fig. 2 graphs, black peptides), 1% milk was better at reducing background compared to any percentage of BSA or 5% milk (Fig. 2g,i, green and blue colored peptides). Thus, we propose performing all blocking steps with 1% milk to reduce background.

### Higher salt washes after reader domain binding decreases non-specific binding events

**Figure 3:**
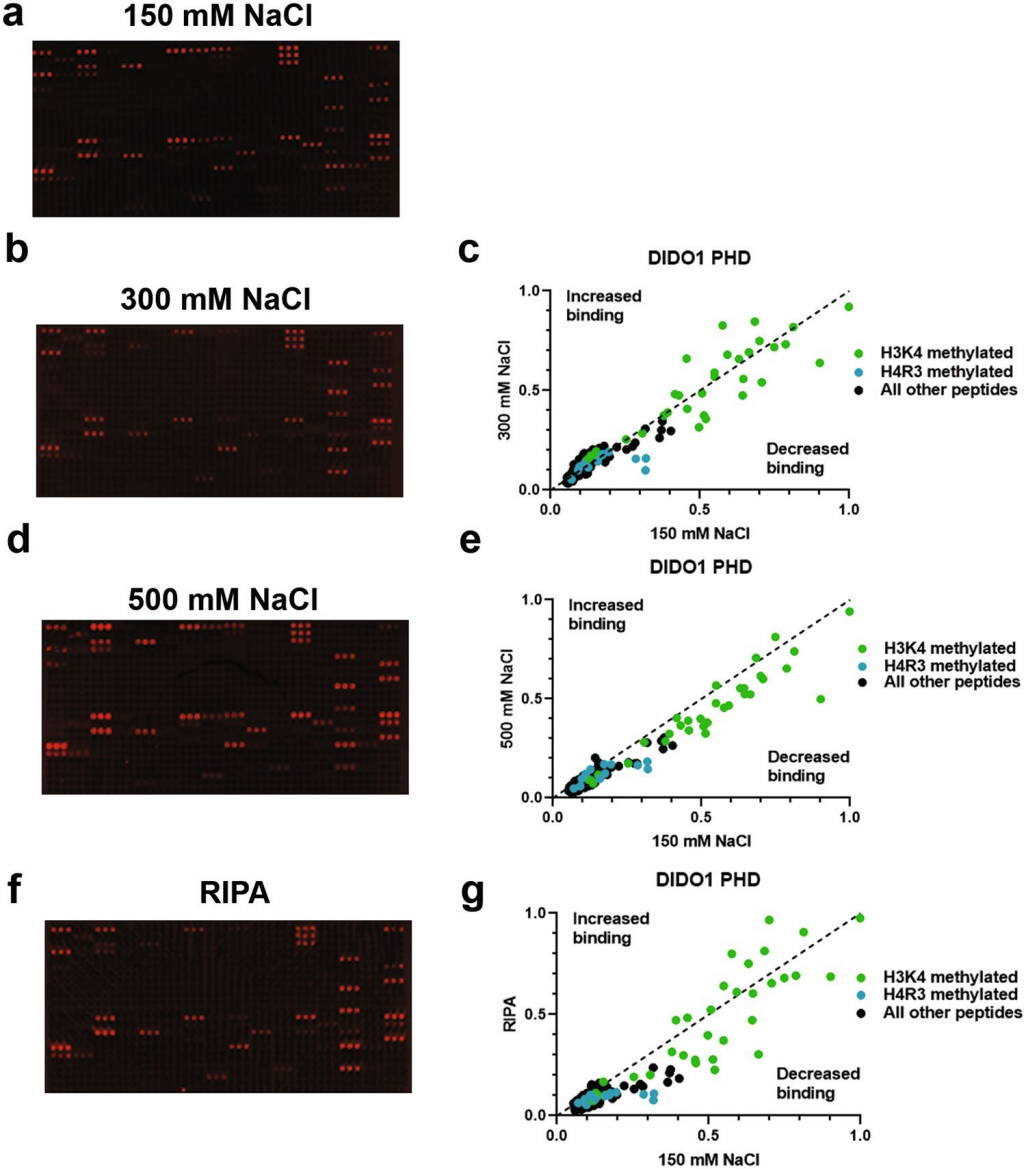
Peptide microarray background is further reduced when slides are washed with 1 X PBS with 500 mM NaCl after domain incubation. (**a–d**) DIDO1 PHD arrays were carried out as in Fig. 2d, with the addition of 3 × 5 minute washes with 1 X PBS with the indicated salt concentration of 150 mM (**a**), 300 mM (**b**), or 500 mM NaCl (**d**) or with RIPA buffer (**f**) after overnight incubation of protein on the domain, which were quantified by normalizing to a 0–1 scale as described in Methods and compared to 1 X PBS (**c, e**, and **g**, respectively). Array intensities were linearly adjusted to equivalent levels and are comparable to each other in the figure. Images are representative of array results for greater or equal to four experiments (i.e., n ≥ 4 subarrays); for additional representative results see Sup. Fig. S2d,f–h. For quantification, averages are shown for each peptide as derived from the replicates of the array results. The average standard deviation for any given peptide was less than 10%.

We next attempted to further reduce the tendency for non-specific binding artifacts to present themselves by altering the post-protein binding wash steps. Given higher salt in the peptide pulldowns assays was successful in reducing non-specific background binding (Fig. 1c), we reasoned that more stringent buffer wash steps in the peptide array procedure might reduce background on the microarrays as well. Thus, we explored the impact of performing our post reader domain-binding washes with 3 × 5-minute washes with 1 X PBS that contained either 150 mM NaCl (original method), 300 mM NaCl, or 500 mM NaCl. We also examined the effects of washing the arrays with standard RIPA buffer. Our tests found that increasing salt concentrations in the washes corresponded to a subtle improvement in the array data, as it further increased the differential between array background and detectable binding (Fig. 3a,b,d and Sup. Fig. S2d,f,g). While the 300 mM NaCl washes had the least effect on outcome, the 500 mM NaCl washes more substantially reduced background; albeit it also reduced the sensitivity of detectable binding as well (Fig. 3b,e). In comparison to NaCl, RIPA buffer, which includes 0.1% SDS, 0.1% Na-deoxycholate, and 150 mM NaCl, also resulted in decreased background but a loss of signal from previously detected hits (Fig. 3f,g and Sup. Fig. S2h). Based on these results, we find that post-binding washes with 500 mM NaCl has the greatest impact on reducing overall array background; yet at the same time subtly reduces the detectable hits. If no other further changes were made to the protocol, 300 mM NaCl salt would be the best overall amount of salt to use in the washes. However, we next sought to determine if the reduced background effects of using high salt might create an advantage in the protocol if we were to first secure reader domains on the arrays.

### Mild cross-linking for a short time period secures low-affinity interactions

**Figure 4:**
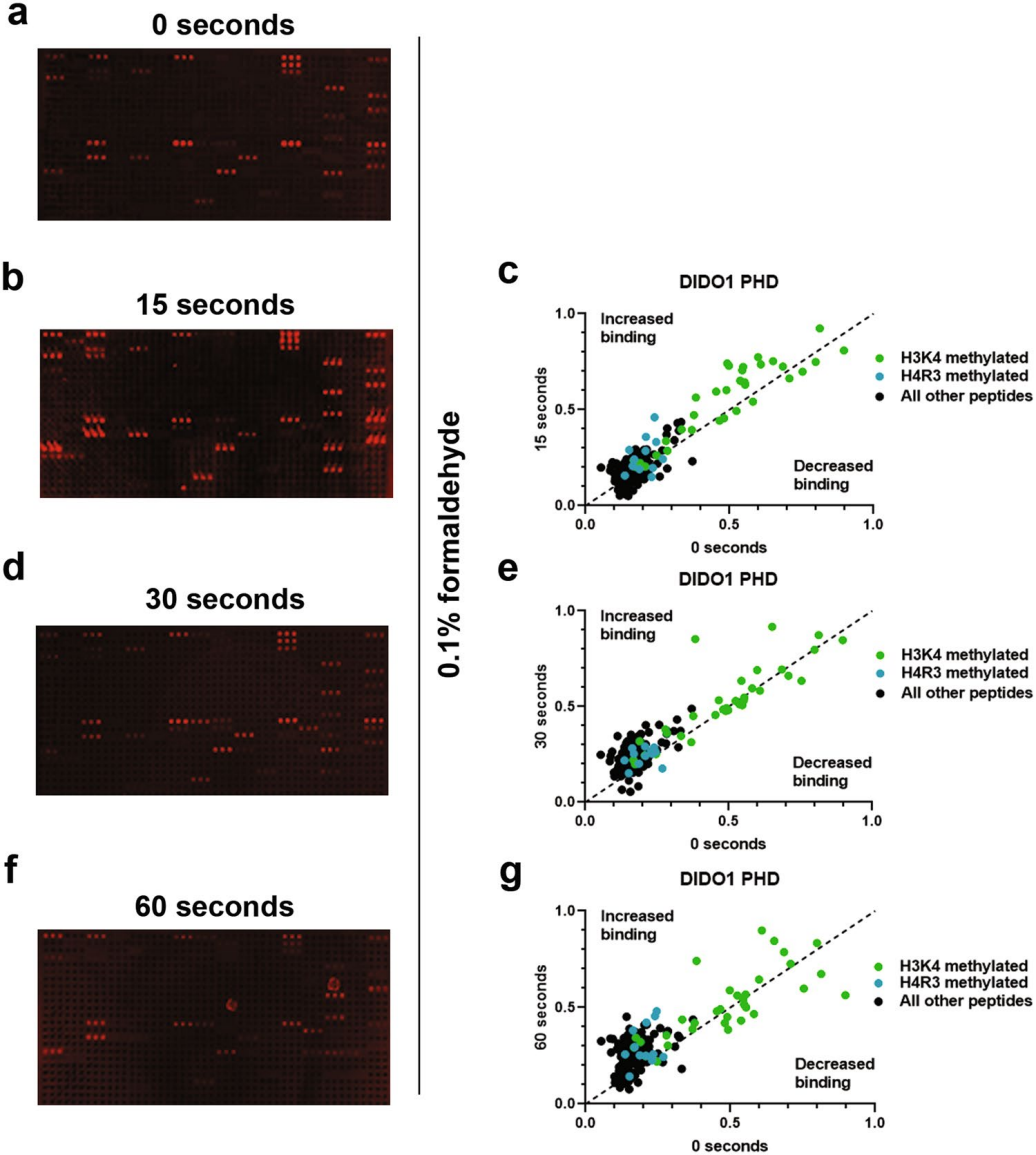
Peptide microarray interactions are better resolved with formaldehyde cross-linking. (**a**) A DIDO1 PHD array without cross-linking, carried out as in Fig. 3c. (**b–d**) DIDO1 PHD array carried out as in Fig. 3c, with a 0.1% formaldehyde in 1 X PBS for the indicated times – 0 seconds (**a**), 15 seconds (**b**), 30 seconds (**d**), and 60 seconds (**f**) – after DIDO1 PHD incubation, followed by quenching with 1 M glycine before 1 X PBS with 500 mM NaCl washes. Arrays were quantified by normalizing to a 0–1 scale as described in Methods and compared to no cross-linking or 0 seconds (**c, e**, and **g**, respectively). Array intensities were linearly adjusted to equivalent levels and are comparable to each other in the figure. Images are representative of array results for greater or equal to four experiments (i.e., n ≥ 4 subarrays); for additional representative results see Sup. Fig. S2g,i-k. For quantification, averages are shown for each peptide as derived from the replicates of the array results. The average standard deviation for any given peptide was less than 10%.

Given that the higher salt washes described above were successful in reducing non-specific binding and the overall background levels, we reasoned that this would be an overall benefit to the microarray procedure if we could first secure the reader domain-peptide interactions. We therefore investigated the use of formaldehyde cross-linking to maintain the protein-peptide interactions prior to the high salt washes. We also surmised that cross-linking might also secure low affinity or more transient protein interactions that are normally lost. We examined the effect of exposing the slides to 0.1% formaldehyde for 15, 30, or 60 seconds prior to quenching for 60 seconds with 1 M glycine. After cross-linking, we proceeded as above- washing 3 × 5 minutes with 1 X PBS at 500 mM NaCl and continuing on with the secondary antibody binding and subsequent steps as described in methods. We found that cross-linking using 0.1% formaldehyde for increasing time periods increased the signal intensity and overall number of signals detected for DIDO1’s PHD finger (Fig. 4 and Sup. Fig. S2g,i–k). Analysis of the array signals for different times versus no time suggested that as time of cross-linking increases, so do signals that were previously weak (blue and green colored peptides); on the other hand, there was a concomitant increase in the non-specific binding events (black peptides) with duration that reduced the ability to establish hits from background (Fig. 4c,e,g). Therefore, we surmise that the optimum amount of formaldehyde and time for cross-linking is 0.1% formaldehyde for 15 seconds, which strikes a balance between the ability to detect weak interactions and prevent the simultaneous probability of promoting spurious binding events.

**Figure 5:**
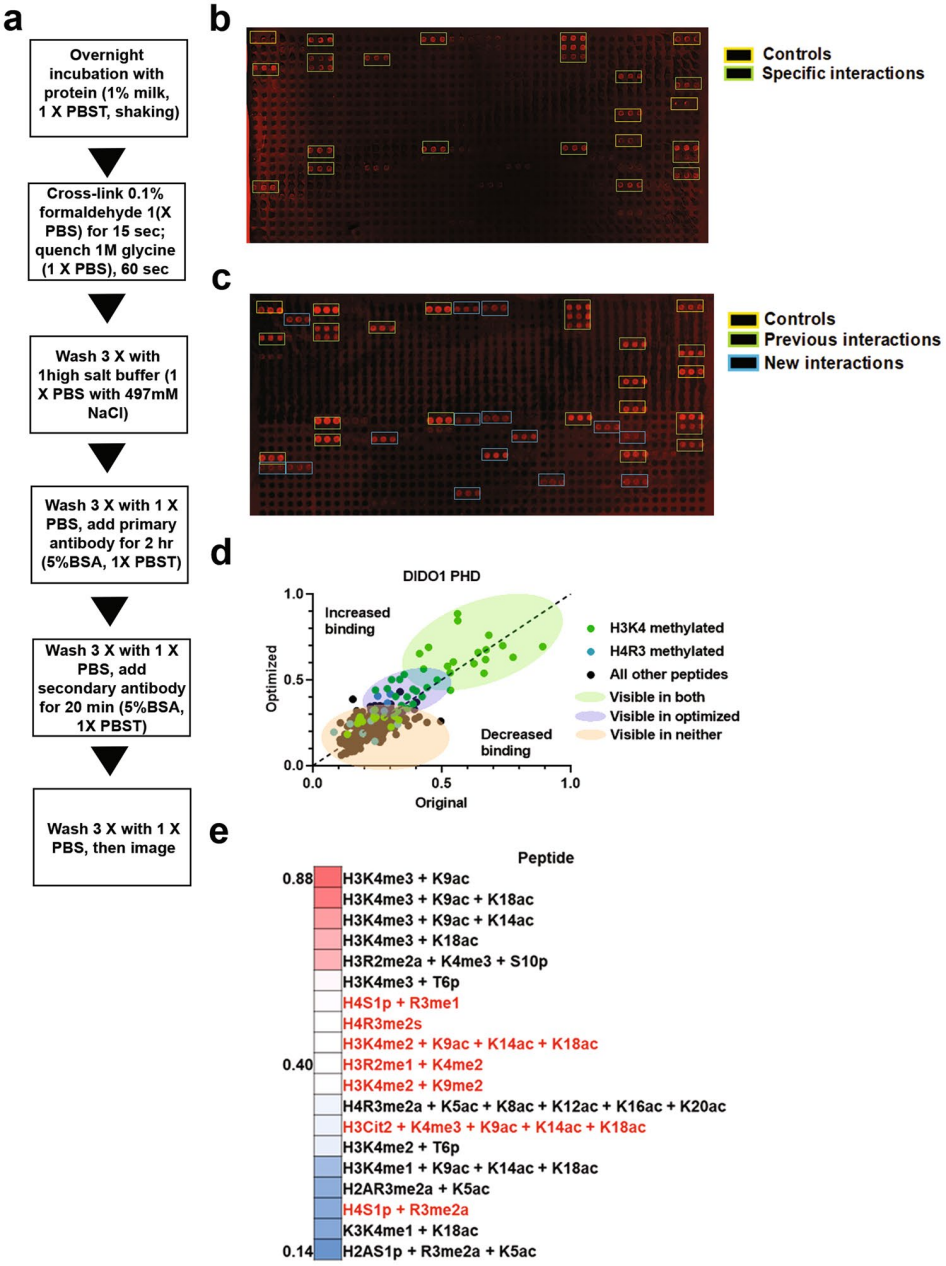
Optimized peptide microarray method increases detectable hits for DIDO1 PHD. (**a**) Flow chart for optimized peptide microarray method. (**b,c**) A DIDO1 PHD peptide microarray using the original (**b**) or optimized (**c**) method, with type of peptides color coded in boxes as indicated. (**d**) Quantification of the DIDO1 PHD results from b and c showing the indicated types of peptides, after data were normalized into a 0 to 1 scale as described in methods and plotted against each other such that values above the dashed line are enhanced by optimization. (**e**) Heatmap diagram of top, middle, and bottom five hits as sorted by values from quantification by the optimized method for the DIDO1 PHD and with new visible hits in red text. Array intensities were linearly adjusted to equivalent levels and are comparable to each other in the figure. Images are representative of array results for greater or equal to four experiments (i.e., n ≥ 4 subarrays) - for additional representative results see Sup. Fig. S2c and S2i. For quantification, averages are shown for each peptide as derived from the replicates of the array results. The average standard deviation for any given peptide was less than 10%.

A direct comparison of the DIDO1 PHD finger-histone interactions using the former and new cross-linking method is presented in Fig. 5, along with a scheme for the optimized protocol (Fig. 5a and Sup. Fig. S2c). For the DIDO1 PHD, the peptides that are discovered with the original method all contain at least the H3K4me3 modification (Fig. 5b and Sup. Fig. S2i). Analysis of the results from the optimized array method revealed novel peptides, including H4R3 methylated, and new H3K4 methylated peptides (Fig. 5c). Quantification of the data revealed that, on average, most of the newly visible peptides that were below or near threshold for detection increased by ~0.2 points on the normalized scale (Fig. 5d,e). Importantly, the peptides that were resolved with the optimized method are consistent with possible interactions indicated by the previous peptide pulldowns (Fig. 1c,e). These data are consistent with the impact of our optimizations on increasing the detection of low affinity interactions and supports the concept that our updates can be applied to other domains.

### The optimized peptide microarray protocol improves detection of weak interactions for other histone-binding domains

**Figure 6:**
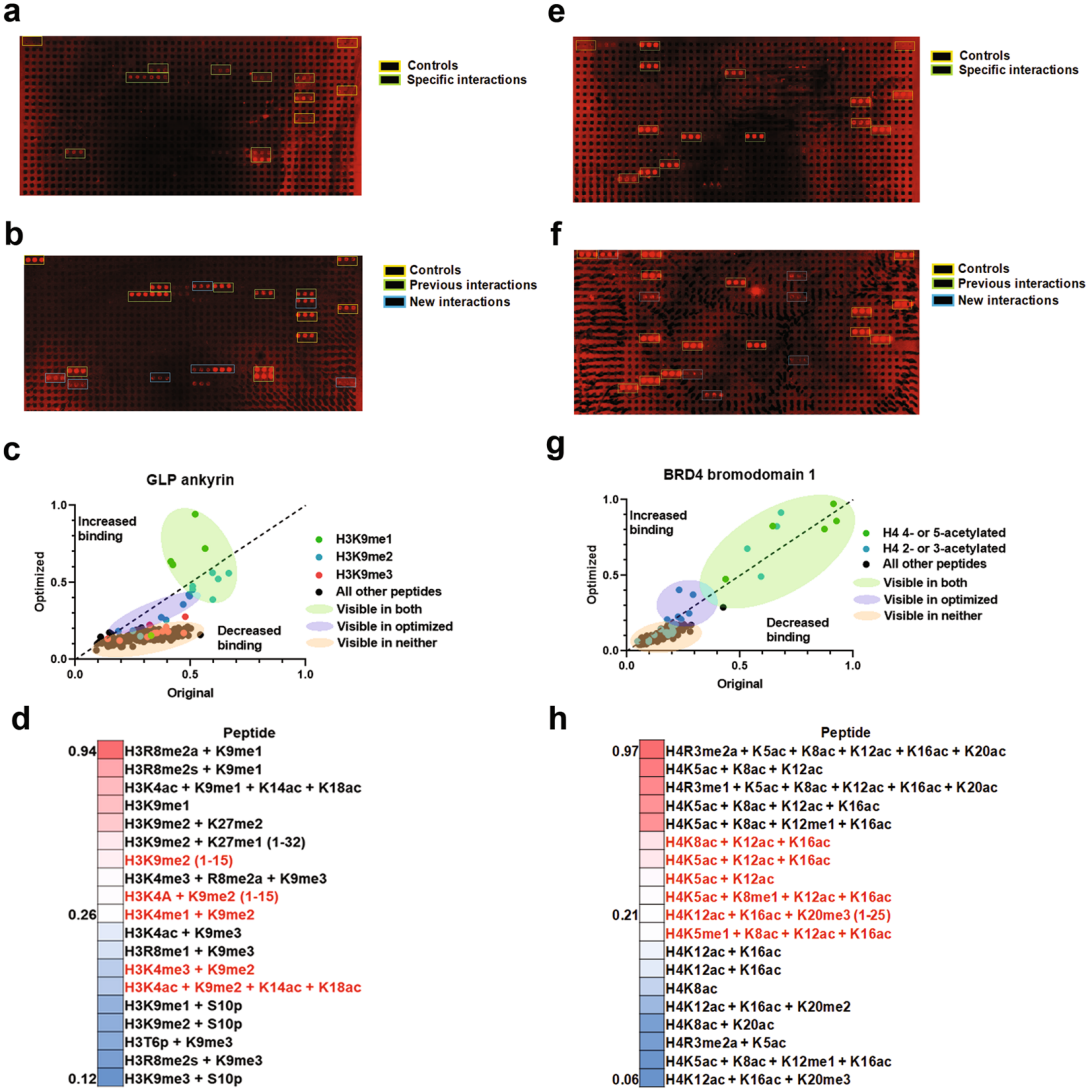
Optimized peptide microarray method increases detectable hits for other reader domains. (**a,b**) A GLP ankyrin peptide microarray using the original (**a**) or optimized (**b**) method, with type of peptides color coded in boxes as indicated. (**c**) Quantification of the G9a ankyrin domain results from a and b showing the indicated types of peptides, after data were normalized into a 0 to 1 scale as described in methods and plotted against each other such that values above the dashed line are enhanced by optimization. (**d**) Heatmap diagram of top, middle, and bottom five hits as sorted by values from quantification by the optimized method for the GLP ankyrin domain and with new visible hits in red text. (**e,f**) BRD4 bromodomain 1 (BD1) peptide microarray using the original (**e**) or optimized (**f**) method, with type of peptides color coded in boxes as indicated. (**g**) Quantification of the BRD4 BD1 results from e and f showing the indicated types of peptides, after data were normalized into a 0 to 1 scale as described in methods and plotted against each other such that values above the dashed line are enhanced by optimization. (**h**) Heatmap diagram of top, middle, and bottom five hits as sorted by values from quantification by the optimized method for BRD4 BD1 and with new visible hits in red text. Array intensities were linearly adjusted to equivalent levels and are comparable to each other in the figure. Images are representative of array results for greater or equal to four experiments (i.e., n ≥ 4 subarrays) - for additional images see Sup. Fig. S3. For quantification, averages are shown for each peptide as derived from the replicates of the array results. The average standard deviation for any given peptide for both domains was less than 10%.

We next asked if our revised peptide microarray protocol would improve the interactions of other reader domains with histones and/or their PTMs. For these analyses, we employed several additional reader domains: the ankryin repeat domain of GLP and the first bromodomain (BD1) of BRD4. While the ankryin repeat domain of GLP interacts predominantly with H3K9 monomethylation, the BD1 of BRD4 interacts with H4 acetylation^22,23^. Initial testing of the ankyrin domain revealed a range of interactions with H3K9me1- and H3K9me2-modified histone peptides when the previous histone peptide microarray protocol is used (Fig. 6a,c,d, and Sup. Fig. S3a,b). Conversely, employing the optimized protocol involving formaldehyde cross-linking and high salt washes resulted in an enhancement of observable hits for peptides carrying H3K9me1 (Fig. 6b–d). Importantly, this is consistent with previous data showing a bias of GLP towards H3K9me1 over H3K9me2 modified peptides^24^. We next compared the effect of the optimized protocol for the acetylation-recognizing BD1 domain of BRD4. Results showed that the signal was greater visually for a number of peptides as compared to the previous protocol (Fig. 6e,f, and Sup. Fig. S3c,d). Similar to the ankyrin repeat, analysis of the performance of the BRD4 BD1 revealed a suppression of background binding and increased detection of peptides that were previously not visible (Fig. 6g,h). Notably, those peptides that were now visible include modifications states with lesser degrees of histone H4 acetylation; specifically, tri- and di-acetylated peptides (Fig. 6h).

Taken as a whole, these data show that the optimized protocol for the peptide microarrays achieves our goal to increase the detectability of weak interactions by both securing the weak affinity binding events and decreasing the background inherent in the arrays. Using the known affinities of BD1 for various individual and combinatorial acetylation states as a reference point to determine the level of improved binding and detection obtained with the new protocol^22,25^, we estimate that the improved protocol increases the range of detection approximately 3–5-fold.

## Discussion

Histone peptide microarrays have been an invaluable tool for the determination of histone PTMs recognition by reader domains and for histone antibody specificity. Because of this importance, we focused on adaptations that would improve the ability to detect interactions that would normally not be readily detected using the standard protocol. Our adjustments, using 1% milk as a blocking agent, introducing a 0.1% formaldehyde cross-linking step for 15 seconds, and then performing a series of 500 mM NaCl washes, form an optimized protocol that is able to resolve interactions that were previously obscured due to high background or loss of weak interactions. This is demonstrated through the analyses of the distinct reader domains found in DIDO1, GLP, and BRD4, which show additional histone peptide interactions that were previously not detectable using the former protocol. This is exemplified with our analysis of DIDO1, where our optimized protocol supports potential new interactions of DIDO1 that are associated with both active and repressive chromatin environments (Figs 1 and 5). This is relevant, since publications support a mechanism where different DIDO1 isoforms can act to toggle between repressed chromatin states and active ones, such as is proposed during embryonic stem cell self-renewal and differentiation^26–28^. It is worth noting that histone peptide microarrays are semi-quantitative and are greatly influenced by the binding capacity of the tested domain, where weak binders tend to return small numbers of hits or fail on the array. However, our data using multiple types of domain suggests that the increase in detection of binding events suggests that within this semi-quantitative framework, our protocol modifications results in a qualitative enhancement in the scope of binding hits returned by peptide microarrays. This is shown through our data for the ankyrin domain of GLP, where the number of H3K9me1 hits was increased but shows no other novel hits, unlike the DIDO1 PHD (Fig. 6). Similarly, bromodomain 1 of BRD4 yielded results in line as an acetyl reader, albeit with additional hits for lesser degrees of poly-acetylation (Fig. 6).

While peptide microarrays are a well-established method for high-throughput screening of peptide-domain interactions, other methods have also been developed. These include mass spectrometry, bead-based assays, and yeast two-hybrid among others^29,30^. Each of these represent their own distinct advantages and disadvantages, which when coupled together or with low-throughput techniques can act orthogonal to each other to validate findings. We consider the ease by which we were able to make the modifications to buffer conditions an indication for the ease and robustness of peptide microarrays a major factor for their continued use, as other methods can require extensive preparatory and detection steps that may not be as readily modified due to constraints^9,10,29,30^. Further, different substrates can be used on which to print peptides. In this study, we used streptavidin coated slides but another common substrate for array fabrication is the SPOT array, which is cellulose based^31,32^. It would be interesting to evaluate if the changes we made herein are amenable with other types of array designs.

The primary goal of these optimizations was to draw out weak binding interactions that were previously occluded for a variety of issues inherent to the array platform and intrinsic affinities of a domain. The modifications we introduced to the standard array protocol were able to largely recapitulate the binding data gained from pulldowns or other methods with greater fidelity as compared to the former protocol, and thereby provide greater insight into interactions that may be biologically relevant. This is more evident when comparing the hits for the tested domains, which suggest that the optimizations allow for the detection of interactions with affinities up to 30 µM. Using known affinities for these domains as a bench mark for how much the arrays have been improved (i.e., the K_d_ of the DIDO PHD with H3K4me3 being ~1 µM versus H3K4me2 being ~6 µM; GLP ankyrin for H3K9me1 being ~5 µM compared to H3K9me2 being ~7 µM; BD1 of being ~3 µM for H4K5ac/K8ac/K12ac/K16ac/K20ac versus ~27 µM for H4K8ac/K12ac, and ~46 µM for H4K12ac/K16ac)^20,24,25^, we find that the modified protocol increases the detection threshold by a factor of ~3–5 in favor of weaker interactions. The potential impact of this becomes evident when considering the large number of reader domains that remain “orphan” readers without known histone targets^33,34^. Significantly, mutations in chromatin interacting domains frequently occur in disease states, underscoring the need to determine their binding preferences for histone modification(s) and how such an interaction impacts the function of the associated protein and/or complex^35,36^. This is particularly relevant, since our optimized method will provide a more complete rendering of the binding possibilities for a given protein, which is critical for understanding the etiology of such epigenetic disorders.

## Methods

### Protein expression and purification

Recombinant proteins were designed and cloned into the pGEX-4T1 bacterial expression system (Amerhsam, 27458001). The GST-tagged proteins were expressed after transformation into SoluBL21 cells (Fisher, C700200) and induced at an OD_600_ of 0.4–0.6 with 1 mM IPTG overnight, ~18 hours, at 16 °C. After induction, cells were lysed, sonicated, and purified as previously described^11^. Briefly, cell pellets were lysed in Lysis Buffer (50 mM HEPES, 150 mM NaCl, 1 mM DTT, 10% glycerol, pH 7.5) with 1x Protease Inhibitors (Sigma, 04693159001), 0.5 mg/mL lysozyme (Sigma, L6876) and 1:20,000 Universal Nuclease (ThermoFisher, PI88700) while incubating on ice for 30 minutes, then sonicated at 45% intensity, 45% duty cycle for 6 × 10 seconds continuously followed by 60 seconds on ice. Lysates were then clarified by centrifugation for 25 minutes at 15,000 rpm. The supernatants were applied to glutathione resin and the GST-tagged proteins were purified by column chromatography according to the manufacturer’s instructions (ThermoFisher, PI16101). The purified, recombinant proteins were then dialyzed into Lysis Buffer to remove residual GSH and stored at −80 °C. Proteins were quantified by Bradford assay according to manufacturer’s instructions (BioRad, 5000006).

### Histone peptide microarrays

Histone peptide microarrays were printed onto glass slides covalently coated with streptavidin (PolyAn, 10402205) as sets of two tandem subarrays per slide, wherein each tandem subarray consists of the same triplicate sets of peptides in different positional order, using a Omnigrid 100 arrayer (Digilab) as described in Rothbart *et al*.^12^. Testing as to the impact of storage conditions revealed that the microarrays are most stable long-term when the slides are stored at −80 °C as compared to 4 °C (data not shown); thus, all microarrays are stored at −80 °C right until their use. To take advantage of both tandem subarrays, each pair of subarrays was segregated with a barrier with the use of a lipid pen (Fisher, NC9545623) that prevented cross-contamination between tandem subarrays and that permitted 500 µL volumes to be used/subarray. The arrays were performed similar to described previously^11^. Briefly, the original and newly optimized hybridization protocols for the histone peptide microarray are as follows:

1. Incubation of the 500 nM protein in 1x PBST (10 mM Na_2_HPO_4_, 1.8 mM KH_2_PO_4_, 2.7 mM KCl, 137 mM NaCl, pH 7.6, 0.1% Tween-20) supplemented with 5% BSA occurred overnight in a humidity chamber, at 4 °C.
2. After binding, the arrays were washed 3 × 5 minutes with 1 X PBS (10 mM Na_2_HPO_4_, 1.8 mM KH_2_PO_4_, 2.7 mM KCl, 137 mM NaCl, pH 7.6) at 4 °C with 1000 rpm shaking.
3. The primary antibody, anti-GST (EpiCypher, 13–0022) at 1:1000, was diluted into 1x PBST with 5% BSA and added onto the array. This was incubated for 2 hours at 4 °C with 1000 rpm shaking.
4. The arrays were washed again 3 × 5 minutes with 1 X PBS at 4 °C with 1000 rpm shaking.
5. The secondary antibody, anti-Rabbit-AlexaFluor 647 (Invitrogen, A21244) at 1:20,000, was also diluted into 1 X PBST with 5% BSA and applied to the array for 30 minutes at 4 °C in the dark with 1000 rpm shaking.
6. A final wash of 1 X PBS for 3 × 5 minutes was performed at 4 °C with 1000 rpm shaking.
7. The arrays were submerged in 0.1 X PBS and blown dry with air, and then imaged with a Typhoon or GenePix Scanner.

The optimized protocol for the histone peptide microarray is as follows:

1. Incubation of the 500 nM protein in 1 X PBST supplemented with 1% non-fat milk occurred overnight in a humidity chamber, at 4 °C with 1000 rpm shaking.
2. Arrays were briefly submerged in 1 X PBS, then submerged in 1 X PBS with 0.1% formaldehyde for 15 seconds, with inversions. The arrays were then transferred and submerged into 1 X PBS with 1 M Glycine for 1 minute, with inversions, to quench the formaldehyde. After quenching, the arrays were submerged in 1 X PBS to remove remaining glycine and formaldehyde by inverting 5×.
3. After cross-linking, the arrays were washed 3 × 5 minutes with high-salt 1 X PBS at 4 °C with 1000 rpm shaking.
4. The primary antibody, anti-GST at 1:1000, was diluted into 1 X PBST with 1% non-fat milk and added onto the array. This was incubated for 2 hours at 4 °C with 1000 rpm shaking.
5. The arrays were washed again 3 × 5 minutes with 1 X PBS at 4 °C with 1000 rpm shaking.
6. The secondary antibody, anti-Rabbit-AlexaFluor 647 at 1:20,000, was also diluted into 1 X PBST with 1% non-fat milk and applied to the array for 30 minutes at 4 °C in the dark with 1000 rpm shaking.
7. A final wash of 1x PBS for 3 × 5 minutes was performed at 4 °C with 1000 rpm shaking.
8. The arrays were submerged in 0.1 X PBS and blown dry with air, and then imaged with a Typhoon or GenePix Scanner.

Modifications to the initial protocol used for the tests to optimize peptide microarrays are further described in the body of the manuscript. For the different washes after cross-linking shown in Fig. 3, high salt buffers are 1 X PBS supplemented with extra NaCl to the indicated concentration (10 mM Na_2_HPO_4_, 1.8 mM KH_2_PO_4_, 2.7 mM KCl, [297 or 497] mM NaCl, pH 7.6) and RIPA (50 mM Tris, 150 mM sodium chloride, 1.0% NP-40, 0.5% sodium deoxycholate, 0.1% SDS, pH 8.0).

Microarrays images were acquired through a Typhoon Trio + (GE) or GenePix 4000B scanner (Axon Instruments) and yielded comparable results. After imaging, the array signals were quantified using ImageQuant TL or GenePix 5.0 software. Analysis of the primary data was carried out as before in Rothbart *et al*.^12^, where the values for each set of triplicates for each subarray were averaged, scaled linearly to a 0–1 range based on the minimum and maximum values for each subarray^12^. The values for each of the subarrays were then averaged together and sorted based on modifications as indicated in the graph legends and supplemental datasets. The average standard deviation for any peptide in the quantifications is indicated in the legend, while the average and standard deviation for each peptide can be found in the file Supplemental Data S1, where the full data sets for each of the three domains can be found. For the experiments comparing original and optimized methods shown in Figs 5 and 6, the rules for visibility above background were defined in part as any peptide with a Z-score ≥ 1 (exact values are reported in Sup. Data. S1), as determined using GraphPad Prism 8.0 software, where the Z-score is defined as: Z = [(|mean-value|)/SD]; however, we also visually inspected each subarray to determine if any peptides called at or below the Z-score were in fact above background by eye (and vice versa) to reduce false calls. Representative images were linearly adjusted such that the background and brightness for all subarrays were equivalent for purposes of visual comparison, and additional representative images are shown in Sup. Figs S2 and S3.

### Peptide pulldowns

Solution peptide pulldowns were performed as described before^11^. Briefly, 50 pmol of protein was incubated with 500 pmol of a biotinylated peptide in Peptide Pulldown Buffer (50 mM Tris, 0.1% NP-40, 0.5% BSA, 150 mM NaCl, pH 8.0) with the indicated concentration of salt at 4 °C with rotation. After 1 hour, 5 µL of streptavidin magnetic beads, which had been blocked with the same Peptide Pulldown Buffer, were added to each peptide pulldown and allowed to interact with the protein and peptide mixture for an additional hour at 4 °C with rotation. After incubation with the magnetic beads, a magnetic rack was used to wash the beads 3 × 5 minutes with Peptide Pulldown Buffer having the same salt concentration as used during binding, while rotating at 4 °C. When washes were completed the supernatant was aspirated and the bound protein was eluted by addition of 50 µL of 1x Laemmli’s SDS Loading Buffer and boiled for 10 minutes at 95 °C prior to loading into a gel. As a control, a lane representing 1% of the input into the pulldown is included; a negative control reaction, where no peptide is present, is processed like those with peptides. The samples were resolved on a 12% SDS-PAGE and transferred to PVDF membrane by semi-dry Western blot, which was blocked after transfer in 1 X TBST (10 mM Tris, 150 mM NaCl, 0.05% Tween-20, pH 7.6) with 5% non-fat dry milk. The primary antibody, anti-GST (EpiCypher, 13–0022) at 1:1000, was incubated overnight in 1 X TBST with 5% non-fat dry milk at 4 °C with rotation. The following day, the blot was washed 3 × 5 minutes in 1 X TBST, then exposed to secondary antibody, anti-Rabbit-HRP at 1:20,000 (GE, NA934V), in 1 X TBST with 5% non-fat dry milk at room temperature with rotation for 1 hour. The blot was washed again 3 times in 1 X TBST, then incubated with chemiluminescent substrate per the manufacturer’s protocol (GE, RPN2232).

## Supplementary information

Supplementary Information

Supplementary Data S1

## Data Availability

All data generated or analyzed during this study are included in this published article (and its Supplementary Information files).
