# Supplementary material for: Improved methods for the detection of histone interactions with peptide microarrays
Source: Sci Rep. 2019 Apr 18;9:6265. doi: 10.1038/s41598-019-42711-y (PMC6472351; doi:10.1038/s41598-019-42711-y)
Supplement: Supplementary file 1 — Supplementary Information [file 41598_2019_42711_MOESM1_ESM.docx]

**Improved methods for the detection of histone interactions with peptide microarrays**

Christopher J. Petell^1,2^, Andrea T. Pham^1^, Jessica Skela^1^, and Brian D. Strahl^1,2^*

^1^Department of Biochemistry and Biophysics, 120 Mason Farm Rd, University of North Carolina at Chapel Hill, NC, USA 27599; USA

^2^UNC Lineberger Comprehensive Cancer Center, 450 West Drive, University of North Carolina at Chapel Hill, NC, USA 27599; USA

*Corresponding Author: (brian_strahl@med.unc.edu)


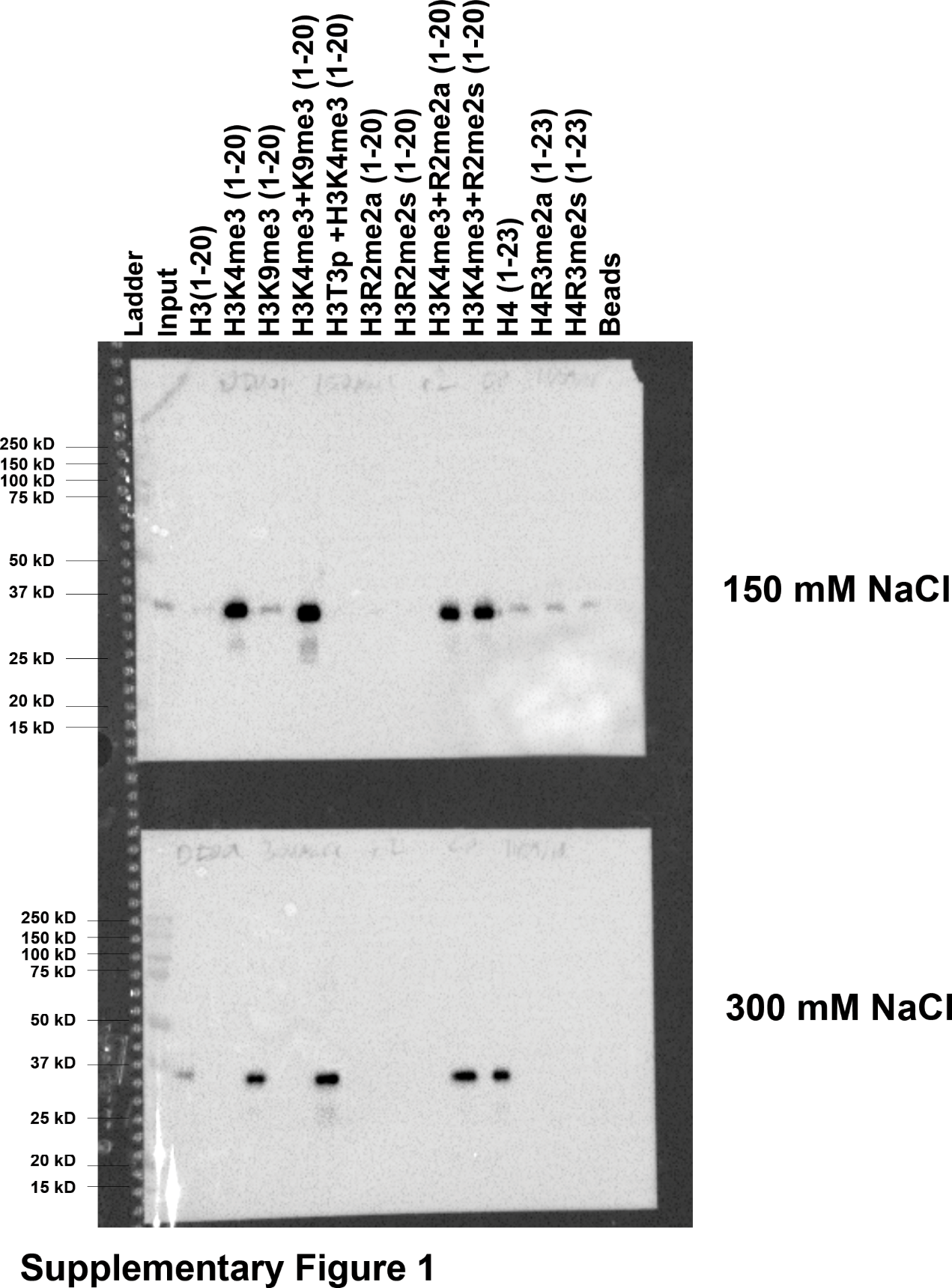


**Supplementary Figure S1: Full images of representative Western blots.**

Uncropped images of the Western blots used in Figure 1c, representative of triplicate experiments.


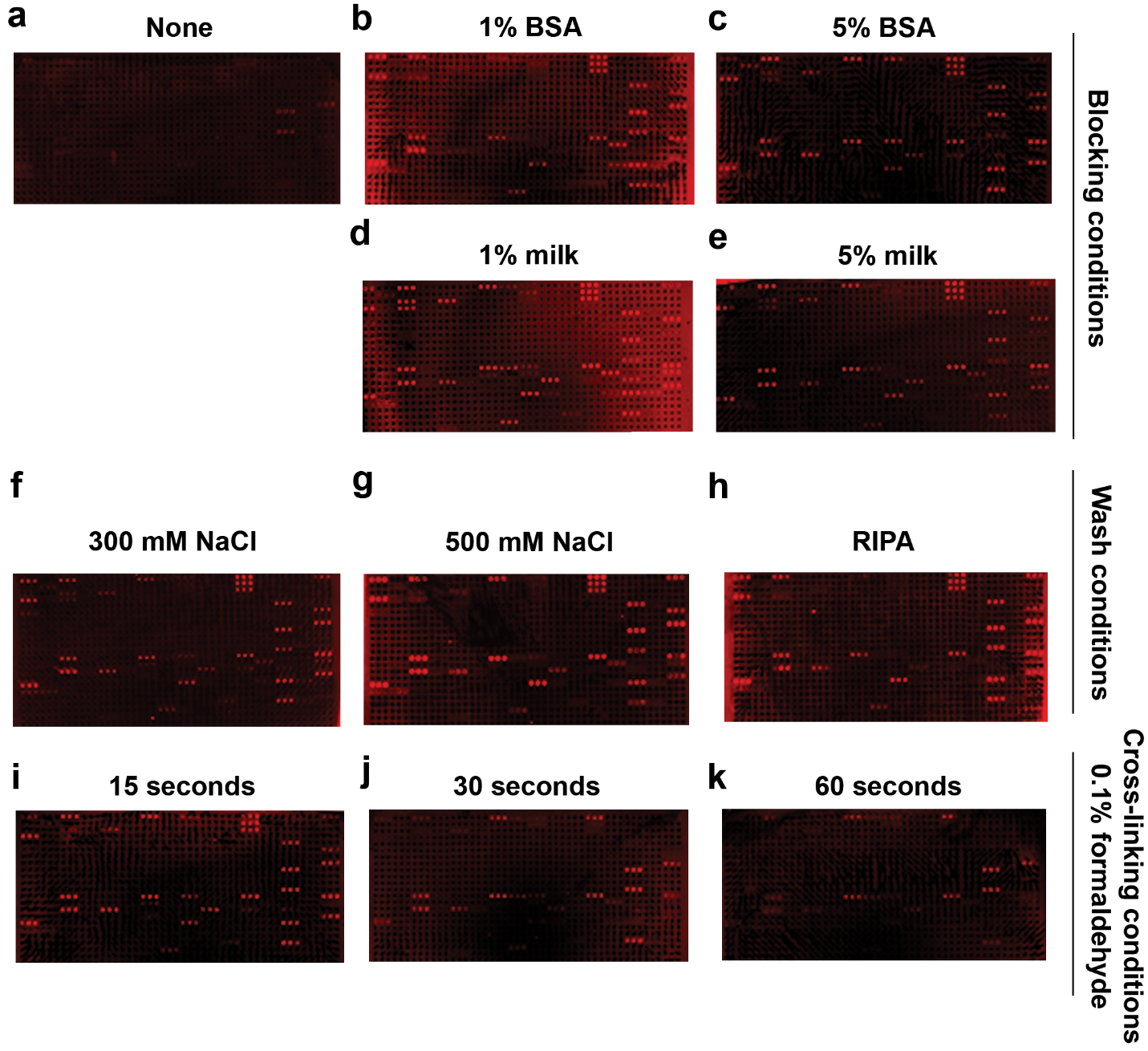


**Supplementary Figure S2: Additional representative images for DIDO1 PHD array experiments.**

a-e) Additional representative images of the arrays presented in Figures 1-2, with panels a-e corresponding to Figure 1b, Figure 2a-d, Figure 3a, and Figure 5b. f-h) Second set of representative images of the arrays presented in Figure 3, where panels f-h correspond to Figure 3b-d and Figure 4a. i-k) Additional set of representative images for arrays shown in Figure 4, with panels i-j corresponding to Figure 4b-d and Figure 5c. Images are representative of array results for greater or equal to four experiments (i.e., n ≥ 4 subarrays).


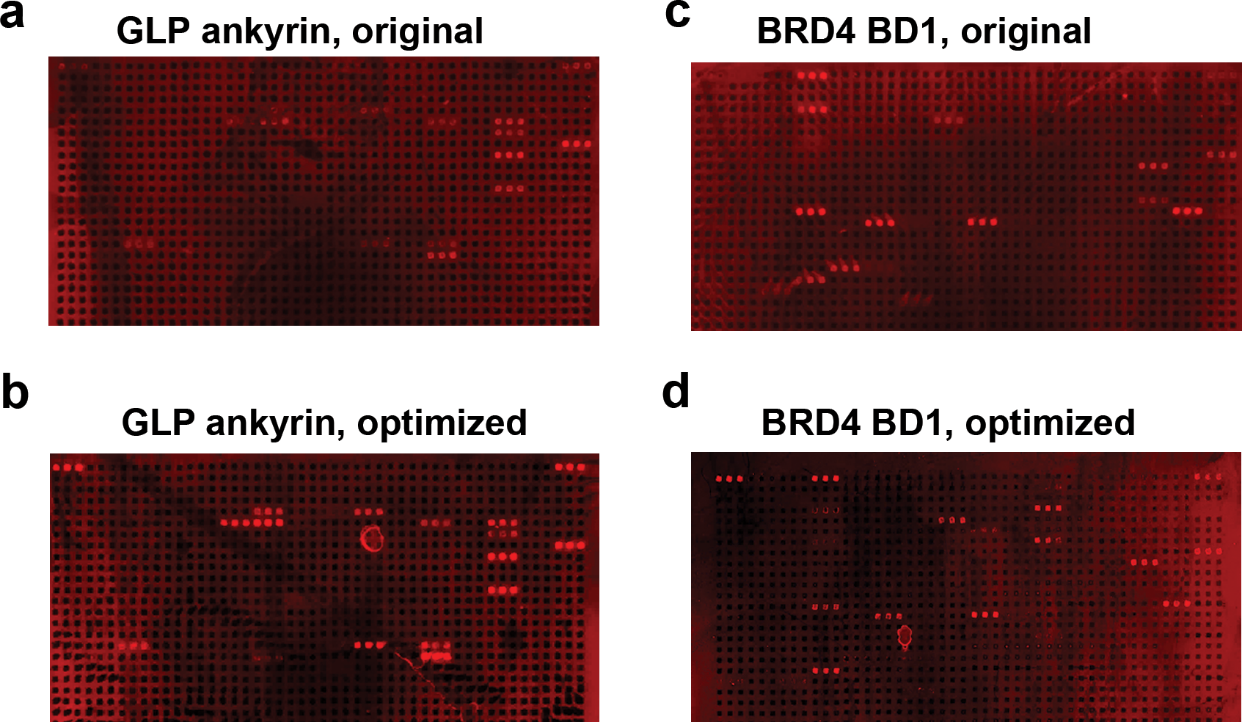


**Supplementary Figure S3: Additional representative images for GLPA ankyrin and BRD4 bromodomain 1 arrays.**

a-d) Additional representative images of the arrays presented in Figure 6 for the GLP ankyrin domain using the original (a) and optimized (b) method and for the BRD4 bromodomain 1 (BD1) using the original c) and optimized (d) method. Images are representative of array results for greater or equal to four experiments (i.e., n ≥ 4 subarrays).
